# Prevalence and determinants of intimate partner violence in pregnancy: a multicentre, binational study

**DOI:** 10.1186/s12884-025-07177-z

**Published:** 2025-02-05

**Authors:** Akaninyene E. Ubom, Chidinma P. Ohachenu, Suraiya S. Auwal, Safiyya F. Usman, Akwasi B. Opoku, Caesar A. Ansing, Jamiu S. Shehu, Peter C. Oriji, Komommo O. Okpebri, Ademola S. Olutoye, Rasheedat O. Balogun, Joshua E. Ifebude, Oluwole D. Obadina, Solomon Nyeche, Abdurrahman A. Bunawa, Ukeje J. Ifeanyi, Fatima A. Mahmud, Hauwa S. Gumbi, Akeem O. Ojugbele, Olubusayo O. Areo, Olakunle E. Ogunjide, Mariam M. Shiru, Ada C. Okpighe, Chia Iornengen, David M. Aqua, Suleiman Z. Abubakar, Fadekemi O. Gabriel-Raji, Oyiana I. Gregory, Lukman O. Lawal, Mathias Abude, David Walawah, Aderopo I. Adelola, Akpofure H. Ese, Jane C. Orijani, Ephraim A. Suobite, Olire C. Afon, Obinna P. Ekwebalam, Baderinwa O. Akanji, Emmanuel E. John, Ibraheem O. Awowole, Omotade A. Ijarotimi, Ngozi Thompson, John I. Ikimalo, Olusola B. Fasubaa

**Affiliations:** 1 Department of Obstetrics, Gynaecology and Perinatology, Obafemi Awolowo University Teaching Hospitals Complex, Ile-Ife, Nigeria; 2 World Association of Trainees in Obstetrics and Gynaecology (WATOG), Paris, France; 3 International Federation of Gynaecology and Obstetrics (FIGO) Committee On Childbirth and Postpartum Haemorrhage, London, United Kingdom; 4 Department of Obstetrics and Gynaecology, Korle-Bu Teaching Hospital, Accra, Ghana; 5 Department of Obstetrics and Gynaecology, National Hospital, Abuja, Nigeria; 6 Department of Obstetrics and Gynaecology, Federal Medical Centre, Abuja, Nigeria; 7 Department of Obstetrics and Gynaecology, Komfo Anokye Teaching Hospital, Kumasi, Ghana; 8 Department of Obstetrics and Gynaecology, Tamale Teaching Hospital, Tamale, Ghana; 9 Department of Obstetrics and Gynaecology, Federal Teaching Hospital, Katsina, Nigeria; 10 Department of Obstetrics and Gynaecology, Federal Medical Centre, Yenagoa, Nigeria; 11 Department of Obstetrics and Gynaecology, University of Calabar Teaching Hospital, Calabar, Nigeria; 12 Department of Obstetrics and Gynaecology, University College Hospital, Ibadan, Nigeria; 13 Department of Obstetrics and Gynaecology, Federal Medical Centre, Lokoja, Nigeria; 14 Department of Obstetrics and Gynaecology, University of Port Harcourt/University of Port Harcourt Teaching Hospital, Port Harcourt, Nigeria; 15 Department of Obstetrics and Gynaecology, Muhammad Abdullahi Wase Teaching Hospital, Kano, Nigeria; 16 Department of Obstetrics and Gynaecology, Federal University Teaching Hospital, Owerri, Nigeria; 17 Department of Obstetrics and Gynaecology, Ahmadu Bello University Teaching Hospital, Zaria, Nigeria; 18 Department of Obstetrics and Gynaecology, University of Ilorin Teaching Hospital, Ilorin, Nigeria; 19 Department of Obstetrics and Gynaecology, Prime Medical Consultants, Port Harcourt, Nigeria; 20 Department of Obstetrics and Gynaecology, University of Nigeria Teaching Hospital, Enugu, Nigeria; 21 Department of Obstetrics and Gynaecology, Modibbo Adama University Teaching Hospital, Yola, Nigeria; 22 Department of Obstetrics and Gynaecology, Lagos University Teaching Hospital, Lagos, Nigeria; 23 Department of Obstetrics and Gynaecology, University of Port Harcourt Teaching Hospital, Port Harcourt, Nigeria; 24 Department of Obstetrics and Gynaecology, Cape Coast Teaching Hospital, Cape Coast, Ghana; 25 Department of Mental Health, Obafemi Awolowo University Teaching Hospitals Complex, Ile-Ife, Nigeria; 26 Department of Obstetrics and Gynaecology, Central Hospital, Warri, Nigeria; 27 Department of Obstetrics and Gynaecology, Jos University Teaching Hospital, Jos, Nigeria; 28 Department of Community Health, Obafemi Awolowo University Teaching Hospitals Complex, Ile-Ife, Nigeria; 29 Department of Obstetrics and Gynaecology, University of Uyo Teaching Hospital, Uyo, Nigeria; 30 Department of Obstetrics, Gynaecology and Perinatology, Faculty of Clinical Sciences, Obafemi Awolowo University, Ile-Ife, Nigeria; 31 Department of Obstetrics and Gynaecology, Ho Teaching Hospital, Ho, Ghana; 32 Department of Obstetrics and Gynaecology, Ahmadu Bello University, Zaria, Nigeria

**Keywords:** IPV, Domestic violence, Domestic abuse, Sub-Saharan Africa

## Abstract

**Background:**

Globally, intimate partners are the most common perpetrators of violence against women. Sub-Saharan Africa (SSA) contributes significantly to the burden of intimate partner violence (IPV) in Africa, with four of every 10 women in SSA having experienced IPV. When IPV occurs in pregnancy, it is significantly associated with adverse outcomes. This study sought to assess the prevalence, determinants and complications of IPV in pregnancy in Nigeria and Ghana, two SSA countries.

**Methods:**

A descriptive, cross-sectional study, conducted between February-July 2022, amongst pregnant women attending antenatal care clinics in 17 health facilities across the six geopolitical zones in Nigeria, and three hospitals in three regions in Ghana. The women were screened for IPV using an adaptation of the ‘HARK’ (Humiliation, Afraid, Rape, Kick) questions. Data obtained were analysed using the IBM SPSS Statistics for Windows, version 25. Associations between IPV and sociodemographic characteristics of the women and their spouses/partners were tested using Pearson’s Chi square. Factors found to be statistically significant were subjected to binary logistic regression modelling to determine the predictors of IPV. The level of statistical significance was set at a *p*-value of < 0.05.

**Results:**

The prevalence of IPV was 26.2%. Predictors of IPV included the women’s marital status (*p* = 0.001), educational status (*p* = 0.040), rural residence (*p* = 0.034), occupation (*p* = 0.040), spouse’s/partner’s occupation (p = 0.021), use of illicit drugs by spouse/partner (*p* = 0.014), history of psychiatric illness in spouse/partner (*p* = 0.030), experience of IPV in previous relationship(s) by spouse/partner (*p* = 0.011), and witness of parental IPV by spouse/partner (*p* < 0.001). The most common complication of IPV in pregnancy were the mental health complications of anxiety (54.7%) and depression (46.9%). Miscarriages (15.6%) and preterm birth (9.5%) were the most common pregnancy complications.

**Conclusions:**

One in every four pregnant women in Nigeria and Ghana experience IPV, with significant mental health and pregnancy complications. Routine IPV screening in pregnancy and prompt referral of screen-positive women to support services is recommended. Policies and interventions that promote gender equality and women’s economic and educational empowerment are beneficial.

**Supplementary Information:**

The online version contains supplementary material available at 10.1186/s12884-025-07177-z.

## Introduction

Intimate partners are the most common perpetrators of violence against women worldwide [1, 2]. Intimate Partner Violence (IPV) encompasses all forms of physical, sexual or emotional/psychological abuse, perpetrated by a current or former partner, within the context of marriage, cohabitation or any other formal or informal union [3]. An estimated 30% of women worldwide have been victims of IPV [4]. In Africa, ever-partnered women have a lifetime risk of physical and/or sexual violence of nearly 40% [4]. Sub-Saharan Africa (SSA) contributes significantly to the burden of IPV in Africa, with an estimated 36%-44.4% of her population affected by IPV [5, 6].

In Nigeria and Ghana, both SSA countries, IPV prevalence rates of over 50% have been reported [7–9]. Findings from previous studies conducted in Nigeria show that at least one in every four Nigerian women have experienced IPV, with emotional violence being the commonest form of IPV reported [7, 10]. In other SSA countries, surveys done in Zambia, Kenya, Uganda and Tanzania, revealed IPV prevalence rates of 40%, 42%, 46% and 60%, respectively [11]. These figures may be underestimations, as many cases of IPV in SSA are unreported, owing to the fact that most affected women view IPV as a family-based problem that should be resolved within the family, without involving third parties. Other women, especially those of low socioeconomic status, are either reluctant or afraid of reporting IPV owing to cultural norms and fear of social stigma [10].

IPV is a serious public health problem that poses significant threat to women’s health and wellbeing globally [2]. Its occurrence in pregnancy is associated with adverse maternal and neonatal outcomes, including spontaneous abortion and antepartum haemorrhage (from abdominal trauma and sexual injury), poor maternal nutrition and weight gain, intrauterine foetal growth restriction, low birth weight, small for gestational age, preterm birth and perinatal deaths [12, 13]. Low birth weight and preterm birth are leading causes of neonatal morbidity and mortality [11]. Violence during pregnancy also increases maternal smoking, alcohol and substance abuse, all of which are significantly associated with inadequate prenatal care and adverse pregnancy and neonatal outcomes [14].

Most of the previous studies on IPV conducted in SSA have focused on IPV in the general population and not specifically in pregnant women. The dearth of studies on IPV in pregnancy in SSA has prevented an extensive exploration of the prevalence and determinants of IPV in pregnancy in the region, hence, many countries in the region lack targeted preventive programs and interventions, with poor awareness of the magnitude of the problem in the general population. This underscores the knowledge and research gaps on IPV in pregnancy in the region. There is, therefore, an urgent need to generate evidence-based information to fill the existing knowledge and research gaps, given the potential magnitude and severity of IPV in pregnancy in SSA countries [11]. More so, the current antenatal care guidelines in both Nigeria and Ghana, the two SSA countries where this study was conducted, do not recommend routine IPV screening in pregnant women. Given that IPV in pregnancy carries a double jeopardy of compromising both the health of the mother and the unborn baby, is there a significant prevalence in these two countries to justify routine screening and support services for IPV in pregnant women? This study seeks to answer this research question. We therefore conducted this multicentre, binational study to determine the prevalence, types, risk factors and complications of IPV in pregnancy in the two SSA countries of Nigeria and Ghana. Pregnancy presents a unique opportunity for vulnerable women to have contact with heath care providers. It is therefore an excellent time to screen for, and detect violence during pregnancy [13]. It is intended that the information generated from this study will guide policies and interventions to mitigate the adverse impacts of IPV in pregnancy in the two countries, and beyond.

## Materials and methods

### Study design and setting

This descriptive cross-sectional study of IPV in pregnancy was conducted over a six-month period, between February-July 2022, amongst pregnant women attending antenatal clinics in 17 randomly selected health facilities (Federal Medical Centres, Teaching Hospitals and Private Hospital) across the six geopolitical zones in Nigeria, and three Teaching Hospitals in three regions in Ghana (Table 1). These hospitals were so selected to obtain nationally representative data from both countries.

**Table 1:** List of health facilities selected for the study

Geopolitical zone/region	Health facilities selected
**Nigeria:**	
North-Central	University of Ilorin Teaching Hospital (UITH), Ilorin, Kwara State
	National Hospital Abuja (NHA)
	Federal Medical Centre (FMC), Abuja
	FMC, Lokoja, Kogi State
North-West	Ahmadu Bello University Teaching Hospital (ABUTH), Zaria, Kaduna State
	Federal Teaching Hospital, Katsina, Katsina State
	Muhammad Abdullahi Wase Teaching Hospital (MAWTH), Kano
North-East	Modibbo Adama University Teaching Hospital, Yola, Adamawa State
South-West	Obafemi Awolowo University Teaching Hospitals Complex (OAUTHC), Ile-Ife, Osun State
	Lagos University Teaching Hospital (LUTH), Lagos State
	University College Hospital (UCH), Ibadan, Oyo State
South-South	University of Port Harcourt Teaching Hospital (UPTH), Port Harcourt, Rivers State
	University of Calabar Teaching Hospital (UCTH), Calabar, Cross Rivers State
	FMC, Yenagoa, Bayelsa State
	Prime Medical Consultants, Port Harcourt, Rivers State
South-East	Federal University Teaching Hospital, Owerri, Imo State
	University of Nigeria Teaching Hospital (UNTH), Enugu State
**Ghana:**	
Greater Accra	Korle Bu Teaching Hospital (KBTH), Accra
Ashanti	Komfo Anokye Teaching Hospital (KATH), Kumasi
Northern	Tamale Teaching Hospital (TTH), Tamale

### Sample size determination and sampling technique

The sample size for this study was calculated using the prevalence study formula: *n* = z^2^ x p x (1-p)/e^2^, where *n* = sample size, z = standard normal variate, which is 1.96 for a confidence level of 95%, *p* = 36% (0.36), which is the IPV prevalence in SSA from a previous study [5], e = margin of error, which is 0.05 at 95% confidence level. Substituting into the formula, a minimum sample size of 355 was obtained. Allowing for a 10% non-response rate, the calculated minimum sample size was approximately 391. Based on antenatal clinic patient volumes, it was estimated that 60% of the study participants would be recruited from the study centers in Nigeria and 40% from the centers in Ghana. Equal numbers of study participants were to be recruited from all the centers in each country. Consecutive sampling was used to recruit all consenting pregnant women who met the inclusion criteria for the study.

### Inclusion criteria

Adult (18 years and above) pregnant women attending antenatal clinics in the aforementioned hospitals, who were married, or if unmarried, in an intimate relationship or cohabiting for at least one year, who gave informed consent to participate in the study.

### Exclusion criteria

Pregnant women who were unmarried and not in an intimate relationship or cohabiting, or unmarried and in an intimate relationship or cohabiting for less than one year, were excluded from the study. Teenage pregnant women who were not up to the age of consent (i.e., 18 years) were also excluded.

### Study instrument and data collection procedure

A 31-item purpose-designed, self-administered questionnaire, containing multiple-choice and open-ended questions, was utilized for the study. The questionnaire collected information on the socio-demographic characteristics of the study participants and their partners/spouses, type of IPV experienced if any, risk factors for IPV, and complications/consequences of IPV. All consenting study participants attending antenatal clinics in the study sites were given the questionnaire, which they completed on their own without any respondent identifying information, and returned to the attending obstetrician afterwards. For illiterate women, health care personnel in the study sites, who were blinded to the study aims and objectives, played the role of language interpreters and read out the questions to the women and the women’s responses to the questions were anonymously recorded. The questionnaire was pretested on 20 pregnant women to ensure reliability and validity before being used for the study.

Study participants were screened for IPV using an adaptation of the ‘HARK’ (Humiliation, Afraid, Rape, Kick) questions, which has been validated and used extensively to screen for IPV [15, 16]. The HARK questions were incorporated in the study questionnaire and included eight questions on the three types/domains of IPV, with options of YES or NO: Psychological/emotional violence- four questions on humiliation or emotional abuse in other ways by spouse/partner and fear of spouse/partner within the last 12 months and during index pregnancy; Sexual violence- two questions on rape or forced sexual activity by spouse/partner within the last 12 months and during index pregnancy; Physical violence- two questions on being kicked, hit, slapped or otherwise physically hurt by spouse/partner within the last 12 months and during index pregnancy (Table 2). A woman was assessed to have experienced a particular type of IPV if she responded positively (answered YES) to any of the questions in that IPV domain.

**Table 2:** Adapted HARK questions

**H**	HUMILIATION
	Within the last 12 months (1 year), have you been humiliated or emotionally abused in other ways by your spouse/partner or ex-spouse/ex-partner?
	During this pregnancy, have you been humiliated or emotionally abused in other ways by your spouse/partner or ex-spouse/ex-partner?
**A**	AFRAID
	Within the last 12 months (1 year), have you been afraid of your spouse/partner or ex-spouse/ex-partner?
	During this pregnancy, have you been afraid of your spouse/partner or ex-spouse/ex-partner?
**R**	RAPE
	Within the last 12 months (1 year), have you been raped or forced to have any kind of sexual activity by your spouse/partner or ex-spouse/ex-partner?
	During this pregnancy, have you been raped or forced to have any kind of sexual activity by your spouse/partner or ex-spouse/ex-partner?
**K**	KICK
	Within the last 12 months (1 year), have you been kicked, hit, slapped, or otherwise physically hurt by your spouse/partner/ex-spouse/ex-partner?
	During this pregnancy, have you been kicked, hit, slapped, or otherwise physically hurt by your spouse/partner or ex-spouse/ex-partner?

### Data analysis

Data obtained was analysed using the IBM Statistical Package for the Social Sciences (SPSS) Statistics for Windows, version 25 (IBM Corp., Armonk, N.Y., USA). Missing data were excluded from analysis. Frequencies and percentages were presented in tables, and associations between IPV and sociodemographic characteristics of the women and their spouses/partners were tested using Pearson’s Chi square. Factors found to be statistically significant were subjected to binary logistic regression modelling to determine the predictors of IPV in the study population. Statistically significant levels were set at *p*-value of < 0.05.

## Results

Within the study period, a total of 958 pregnant women were recruited: 572 (59.7%) of these were recruited from Nigeria and 386 (40.3%) from Ghana.

### Sociodemographic and obstetric characteristics of the study participants

The mean age and gestational age of the women were 30.0 ± 5.6 years and 28.4 ± 8.7 weeks, respectively. The median (IQR) parity was 2 (1–3). Most of the women were married (808/950, 85.1%), professionals (361/940, 38.4%), with tertiary education (574/939, 61.1%) and urban residence (656/936, 70.1%) (Table 3).

**Table 3:** Sociodemographic and obstetrics characteristics of the study participants

Characteristic	All women (%)	Nigeria (%)	Ghana (%)
**Age (years)**	***n* = 944**	***n* = 563**	***n* = 381**
< 20	23 (2.4)	11 (2.0)	12 (3.1)
20–29	434 (46.0)	266 (47.2)	168 (44.1)
30–39	439 (46.5)	255 (45.3)	184 (48.3)
≥ 40	48 (5.1)	31 (5.5)	17 (4.5)
**Marital status**	***n* = 950**	***n* = 566**	***n* = 384**
Single/unmarried but co-habiting	122 (12.8)	33 (5.8)	89 (23.2)
Married	808 (85.1)	519 (91.7)	289 (75.3)
Divorced/Separated	20 (2.1)	14 (2.5)	6 (1.6)
**Level of Education**	***n* = 939**	***n* = 556**	***n* = 383**
No formal education	39 (4.2)	8 (1.4)	31 (8.1)
Primary	74 (7.9)	29 (5.2)	45 (11.7)
Secondary	252 (26.8)	104 (18.7)	148 (38.6)
Tertiary	574 (61.1)	415 (74.6)	159 (41.5)
**Residence**	***n* = 936**	***n* = 555**	***n* = 381**
Rural	100 (10.7)	64 (11.5)	36 (9.4)
Semi-urban	180 (19.2)	95 (17.1)	85 (22.3)
Urban	656 (70.1)	396 (71.4)	260 (68.2)
**Occupation**	***n* = 940**	***n* = 564**	***n* = 376**
Professional	361 (38.4)	260 (46.1)	101 (26.9)
Skilled worker	265 (28.2)	124 (22.0)	141 (37.5)
Unskilled worker	134 (14.3)	77 (13.7)	57 (15.2)
Unemployed	180 (19.1)	103 (18.3)	77 (20.5)
**Parity**	***n* = 879**	***n* = 546**	***n* = 333**
0	229 (26.1)	127 (23.3)	102 (30.6)
1–4	602 (68.5)	371 (67.9)	231 (69.4)
≥ 5	48 (5.5)	48 (8.8)	0 (0)
**Mean EGA ± SD (weeks)**	28.4 ± 8.7	27.1 ± 8.6	30.3 ± 8.5

n ≠ 958, 572 and 386 owing to missing data, which were excluded from analysis

### Sociodemographic characteristics of the study participants’ spouses/partners

The mean age of the women’s spouses/partners was 36.6 ± 6.8 years, which was significantly higher than the mean age of the women (*p* < 0.001). Majority of them were professionals (430/922, 46.6%), with tertiary education (639/945, 67.6%). Less than one-tenth of them smoked cigarettes (67/907, 7.4%) or took illicit drugs (35/881, 4.0%). About a tenth (67/868, 7.7%) had witnessed IPV between their parents growing up. Only 36/907, 4.0%, and 32/905, 3.5%, respectively, had history of psychiatric illness and had been victims of IPV in previous relationships. These sociodemographic characteristics are shown in Table 4.

**Table 4:** Sociodemographic characteristics of the study participants’ spouses/partners

Characteristic	All women (%)	Nigeria (%)	Ghana (%)
**Age (years)**	***n* = 936**	***n* = 559**	***n* = 377**
< 20	3 (0.3)	0 (0)	3 (0.8)
20–29	112 (12.0)	49 (8.8)	63 (16.7)
30–39	503 (53.7)	303 (54.2)	200 (53.1)
≥ 40	318 (34.0)	207 (37.0)	111 (29.4)
**Level of Education**	***n* = 945**	***n* = 563**	***n* = 382**
No formal education	26 (2.8)	11 (2.0)	15 (3.9)
Primary	47 (5.0)	13 (2.3)	34 (8.9)
Secondary	233 (24.7)	104 (18.5)	129 (33.8)
Tertiary	639 (67.6)	435 (77.3)	204 (53.4)
**Occupation**	***n* = 922**	***n* = 543**	***n* = 379**
Professional	430 (46.6)	301 (55.4)	129 (34.0)
Skilled worker	308 (33.4)	155 (28.5)	153 (40.4)
Unskilled worker	141 (15.3)	68 (12.5)	73 (19.3)
Unemployed	43 (4.7)	19 (3.5)	24 (6.3)
**Smoking of cigarette**	***n* = 907**	***n* = 524**	***n* = 383**
Yes	67 (7.4)	45 (8.6)	22 (5.7)
No	840 (92.6)	479 (91.4)	361 (94.3)
**Use of illicit drugs**	***n* = 881**	***n* = 502**	***n* = 379**
Yes	35 (4.0)	22 (4.4)	13 (3.4)
No	846 (96.0)	480 (95.6)	366 (96.6)
**History of psychiatric illness**	***n* = 907**	***n* = 530**	***n* = 377**
Yes	36 (4.0)	25 (4.7)	11 (2.9)
No	821 (90.5)	455 (85.8)	366 (97.1)
I don’t know	50 (5.5)	50 (9.4)	0 (0)
**Victim of IPV in previous relationship(s)**	***n* = 905**	***n* = 532**	***n* = 373**
Yes	32 (3.5)	27 (5.1)	5 (1.3)
No	815 (90.1)	447 (84.0)	368 (98.7)
I don’t know	58 (6.4)	58 (10.9)	0 (0)
**Witnessed parental IPV**	***n* = 868**	***n* = 497**	***n* = 371**
Yes	67 (7.7)	33 (6.6)	34 (9.2)
No	747 (86.1)	410 (82.5)	337 (90.8)
I don’t know	54 (6.2)	54 (10.9)	0 (0)

n ≠ 958, 572 and 386 owing to missing data, which were excluded from analysis

### Prevalence and types of IPV in pregnancy in the study population

The overall prevalence of IPV in the study population was 26.2% (229/873), with comparative prevalence in Nigeria and Ghana of 27.7% (135/488) and 24.4% (94/385), respectively. The most common type of IPV in this study was psychological/emotional violence, suffered by 24.6% (215/875) of the women, while sexual violence (71/874, 8.1%) was the least reported type of IPV. Similarly, in both countries, psychological/emotional violence was commonest, reported by 26.1% (128/490) of the women in Nigeria and 22.6% (87/385) of those in Ghana. Sexual violence was also the least common type of IPV in both Nigeria (46/489, 9.4%) and Ghana (25/385, 6.5%), as shown in Table 5.

**Table 5:** Prevalence and types of IPV in pregnancy among the study population

Variable	All women (%)	Nigeria (%)	Ghana (%)
**Suffered IPV within the last 12 months/in index pregnancy**	*n* = 873	*n* = 488	*n* = 385
Yes	229 (26.2)	135 (27.7)	94 (24.4)
No	644 (73.8)	353 (72.3)	291 (75.6)
**Type of IPV suffered**			*n* = 385
Psychological/emotional violence	215/875 (24.6)	128/490 (26.1)	87 (22.6)
Physical violence	102/874 (11.7)	59/489 (12.1)	43 (11.2)
Sexual violence	71/874 (8.1)	46/489 (9.4)	25 (6.5)
**Psychological/emotional violence**			
*Humiliation or emotional abuse in other ways by spouse/partner within the last 12 months*	*n* = *868*	*n* = *484*	*n* = *384*
Yes	168 (19.4)	102 (21.1)	66 (17.2)
No	700 (80.6)	382 (78.9)	318 (82.8)
*Humiliation or emotional abuse in other ways by spouse/partner in index pregnancy*	*n* = *866*	*n* = *482*	*n* = *384*
Yes	153 (17.7)	89 (18.5)	64 (16.7)
No	713 (82.3)	393 (81.5)	320 (83.3)
*Afraid of spouse/partner within the last 12 months*	*n* = *869*	*n* = *487*	*n* = *382*
Yes	126 (14.5)	75 (15.4)	51 (13.4)
No	743 (85.5)	412 (84.6)	331 (86.6)
*Afraid of spouse/partner in index pregnancy*	*n* = *869*	*n* = *486*	*n* = *383*
Yes	116 (13.3)	65 (13.4)	51 (13.3)
No	753 (86.7)	421 (86.6)	332 (86.7)
**Sexual violence**			
*Rape or forced sexual activity by spouse/partner within the last 12 months*	*n* = *873*	*n* = *488*	*n* = *385*
Yes	53 (6.1)	31 (6.4)	22 (5.7)
No	820 (93.9)	457 (93.6)	363 (94.3)
*Rape or forced sexual activity by spouse/partner in index pregnancy*	*n* = *865*	*n* = *484*	*n* = *381*
Yes	46 (5.3)	29 (6.0)	17 (4.5)
No	819 (94.7)	455 (94.0)	364 (95.5)
**Physical violence**			
*Kicked, hit, slapped, or otherwise physically hurt by spouse/partner within the last 12 months*	*n* = *868*	*n* = *487*	*n* = *381*
Yes	92 (10.6)	53 (10.9)	39 (10.2)
No	776 (89.4)	434 (89.1)	342 (89.8)
*Kicked, hit, slapped, or otherwise physically hurt by spouse/partner in index pregnancy*	*n* = *860*	*n* = *480*	*n* = *380*
Yes	61 (7.1)	39 (8.1)	22 (5.8)
No	799 (92.9)	441 (91.9)	358 (94.2)

n ≠ 958, 572 and 386 owing to missing data, which were excluded from analysis

### Complications of IPV in pregnancy and IPV report by study participants

More than three-fourth (179/229, 78.2%) of the women who suffered IPV reported complications. The most common complications were the mental health complications of anxiety (98/179, 54.7%) and depression (84/179, 46.9%). Nearly one-tenth (15/179, 8.3%) of the IPV victims had suicidal ideation or had attempted suicide. Miscarriages (28/179, 15.6%) and preterm birth (17/179, 9.5%) were the most commonly reported pregnancy complications, while almost a third (56/179, 31.3%) of the victims reported marital disharmony, reduced care and affection and sex denial, leading to separation/divorce in 29/179, 16.2%, of them (Table 6).

**Table 6:** Complications of IPV in pregnancy and IPV report by study participants

Variable	All women (%)	Nigeria (%)	Ghana (%)
**IPV complication in index/previous pregnancy**	*n* = 229	*n* = 135	*n* = 94
Yes	179 (78.2)	107 (79.3)	72 (76.6)
No	50 (21.8)	28 (20.7)	22 (23.4)
**Type of IPV complication**^a^	*n* = 179	*n* = 107	*n* = 72
*Pregnancy complications*			
Miscarriage	28 (15.6)	17 (15.9)	11 (15.3)
Preterm birth	17 (9.5)	11 (10.3)	6 (8.3)
IUFD/stillbirth	4 (2.2)	2 (1.9)	2 (2.8)
Abruptio placentae	2 (1.1)	2 (1.9)	-
*Psychological/mental health complications*			
Anxiety	98 (54.7)	59 (55.1)	39 (54.2)
Depression	84 (46.9)	54 (50.5)	30 (41.7)
Panic disorder	32 (17.9)	21 (19.6)	11 (15.3)
Suicidal ideation/attempt	15 (8.3)	6 (5.6)	9 (12.5)
Deliberate self-harm	13 (7.3)	9 (8.4)	4 (5.6)
Sadness/mood swings	2 (1.1)	2 (1.9)	-
*Marital problems*			
Marital disharmony/reduced care and affection/sex denial	56 (31.3)	37 (34.6)	19 (26.4)
Separation/divorce	29 (16.2)	9 (8.4)	20 (27.8)
**Reported IPV to anyone**	*n* = 191	*n* = 117	*n* = 74
Yes	91 (47.6)	57 (48.7)	34 (45.9)
No	100 (52.4)	60 (51.3)	40 (53.4)
**If Yes, reported to who?**	*n* = 91^a^	*n* = 57^a^	*n* = 34
Family/relatives/friends	86 (94.5)	57 (100.0)	29 (85.3)
Social welfare department	5 (5.5)	4 (7.0)	1 (2.9)
The police	5 (5.5)	2 (3.5)	3 (8.8)
Religious group/spiritual leaders	3 (3.3)	2 (3.5)	1 (2.9)
**If No, why?**	*n* = 93	*n* = 57	*n* = 36
Did not want a third party to be aware	32 (34.4)	22 (38.6)	10 (27.8)
Afraid to report	24 (25.8)	13 (22.8)	11 (30.6)
Threatened by spouse not to report	12 (12.9)	8 (14.0)	4 (11.1)
Did not deem it necessary to report/did not have trusted friends/felt she could handle it on her own	3 (3.2)	1 (1.8)	2 (5.6)
No reason	22 (23.7)	13 (22.8)	9 (25.0)

*IUFD* intrauterine foetal death

^a^Multiple responses, hence n > 179, 107, 72, 91, 57

More than one-half (100/191, 52.4%) of the IPV victims did not report the violence, with two-third (56/93, 60.2%) of them citing that they did not report either because they did not want a third party to be aware or were afraid to report. Of those who reported, an overwhelming majority (86/91, 94.5%) of them reported to their families, relatives and friends. Only a tenth (10/91, 11.0%) of IPV victims reported to the social welfare department or police (Table 6).

### Association between IPV in pregnancy and the women’s and their spouses’/partners’ sociodemographic and obstetric characteristics

On bivariate analysis, maternal sociodemographic characteristics that were significantly associated with IPV in pregnancy included: young age < 30 years (*p* = 0.045), separated/divorced marital status (*p* < 0.001), illiterate/low educational status (*p* < 0.001), rural residence (*p* < 0.001), and being unskilled/unemployed (*p* < 0.001). Parity showed no association with IPV in pregnancy (*p* = 0.280) (Table 7).

**Table 7:** Association between IPV in pregnancy and the women’s sociodemographic characteristics

Characteristic	IPV		*p*-value
	Yes (%)	No (%)	
**Age (years)**			
< 30 (*n* = 424)	125 (29.5)	299 (70.5)	0.045^*^
≥ 30 (*n* = 435)	102 (23.4)	333 (76.6)	
**Marital Status**			
Single/unmarried but co-habiting (*n* = 122)	36 (29.5)	86 (70.5)	< 0.001^*^
Married (*n* = 723)	176 (24.3)	547 (75.7)	
Separated/Divorced (*n* = 20)	15 (75.0)	5 (25.0)	
**Level of Education**			
No formal education (*n* = 39)	15 (38.5)	24 (61.5)	< 0.001^*^
Primary/secondary (*n* = 317)	113 (35.6)	204 (64.4)	
Tertiary (*n* = 498)	95 (19.1)	403 (80.9)	
**Residence**			
Rural (*n* = 90)	38 (42.2)	52 (57.8)	< 0.001^*^
Semi-urban/urban (*n* = 759)	186 (24.5)	573 (75.5)	
**Occupation**			
Professional (*n* = 304)	50 (16.4)	254 (83.6)	< 0.001*
Skilled worker (*n* = 253)	63 (24.9)	190 (75.1)	
Unskilled worker/unemployed (*n* = 294)	111 (37.8)	183 (62.2)	
**Parity**			
Nullipara (*n* = 216)	50 (23.1)	166 (76.9)	0.280
Multipara (*n* = 587)	158 (26.9)	429 (73.1)	

^*^Statistical significance. 30 years was used as the age cut-off being the mean age of the women

Spousal/partner risk factors that were significantly associated with IPV in pregnancy were age ≥ 40 years (*p* = 0.044), illiterate/low educational status (*p* < 0.001), unskilled/unemployed status (*p* < 0.001), smoking (*p* < 0.001), illicit drug use (*p* < 0.001), current or past history of psychiatric illness (*p* < 0.001), being a victim of IPV in a previous relationship(s) (*p* < 0.001) and having witnessed parental IPV (*p* < 0.001) (Table 8).

**Table 8:** Association between IPV in pregnancy and the women’s spouse’s/partner’s sociodemographic characteristics

Characteristic	IPV		*p*-value
	Yes (%)	No (%)	
**Age (years)**			
< 40 (*n* = 571)	145 (25.4)	426 (74.6)	0.044^*^
≥ 40 (*n* = 280)	82 (29.3)	198 (70.7)	
**Level of Education**			
No formal education (*n* = 24)	12 (50.0)	12 (50.0)	< 0.001^*^
Primary/secondary (*n* = 271)	93 (34.3)	178 (65.7)	
Tertiary (*n* = 566)	121 (21.4)	445 (78.6)	
**Occupation**			
Professional (*n* = 370)	73 (19.7)	297 (80.3)	< 0.001^*^
Skilled worker (*n* = 286)	76 (26.6)	210 (73.4)	
Unskilled worker/unemployed (*n* = 180)	73 (40.6)	107 (59.4)	
**Smoking**			
Yes (*n* = 64)	43 (67.2)	21 (32.8)	< 0.001^*^
No (*n* = 785)	176 (22.4)	609 (77.6)	
**Illicit drug use**			
Yes (*n* = 33)	26 (78.8)	7 (21.2)	< 0.001^*^
No (*n* = 790)	185 (23.4)	605 (76.6)	
**History of psychiatric illness**			
Yes (*n* = 35)	26 (74.3)	9 (25.7)	< 0.001^*^
No (*n* = 764)	172 (22.5)	592 (77.5)	
**Victim of IPV in previous relationship(s)**			
Yes (*n* = 30)	22 (73.3)	8 (26.7)	< 0.001^*^
No (*n* = 758)	164 (21.6)	594 (78.4)	
**Witnessed parental IPV**			
Yes (*n* = 67)	51 (76.1)	16 (23.9)	< 0.001^*^
No (*n* = 690)	127 (18.4)	563 (81.6)	

^*^Statistical significance. 40 years was used as the age cut-off being the approximate mean age

### Predictors of IPV in pregnancy in the study population

Binary logistic regression modelling showed that the woman’s marital status (*p* = 0.001), educational status (*p* = 0.040), place of residence (*p* = 0.034), occupation (*p* = 0.040), spouse’s/partner’s occupation (*p* = 0.021), use of illicit drugs by spouse/partner (*p* = 0.014), history of psychiatric illness in spouse/partner (*p* = 0.030), experience of IPV in previous relationship(s) by spouse/partner (*p* = 0.011), and witness of parental IPV by spouse/partner (*p* < 0.001) were predictors of IPV in pregnancy in the study population.

Women who were married (OR = 0.076, 95% CI: 0.020–0.283, *p* < 0.001) or single/unmarried but co-habiting (OR = 0.090, 95% CI: 0.022–0.360, *p* = 0.001), and professionals (OR = 0.536, 95% CI: 0.326–0.879, *p* = 0.014) were less likely to experience IPV in pregnancy compared to those who were separated/divorced and unskilled/unemployed, respectively (Table 9). Those who were primary/secondary school leavers (OR = 1.678, 95% CI: 1.119–2.518, *p* = 0.012) and who resided in rural areas (OR = 1.767, 95% CI: 1.043–2.994, *p* = 0.034) were twice more likely to experience IPV compared to women who had tertiary education and resided in semi-urban/urban settings (Table 9).

**Table 9:** Predictors of IPV in pregnancy in the study population

Variable	Odds Ratio (OR)	95% CI	*p*-value
**Woman’s age (years)**			
≥ 30	1.000	Ref	Ref
< 30	1.023	0.706–1.483	0.905
**Spouse’/partner’s age (years)**			
≥ 40	1.000	Ref	Ref
< 40	0.885	0.571–1.373	0.586
**Woman’s marital status**			
Divorced/ Separated	1.000	Ref	Ref
Married	0.076	0.020–0.283	< 0.001^*^
Single/unmarried but cohabiting	0.090	0.022–0.360	0.001^*^
**Woman’s level of education**			
Tertiary	1.000	Ref	Ref
No formal education	1.657	0.716–3.837	0.238
Primary/secondary	1.678	1.119–2.518	0.012^*^
**Spouse’s/partner’s level of education**			
Tertiary	1.000	Ref	Ref
No formal education	1.564	0.508–4.818	0.436
Primary/secondary	1.524	0.938–2.476	0.089
**Woman’s residence**			
Semi-urban/urban	1.000	Ref	Ref
Rural	1.767	1.043–2.994	0.034^*^
**Woman’s occupation**			
Unskilled/unemployed	1.000	Ref	Ref
Professional	0.536	0.326–0.879	0.014^*^
Skilled worker	0.700	0.454–1.079	0.106
**Spouse’s/partner’s occupation**			
Unskilled/unemployed	1.000	Ref	Ref
Professional	0.463	0.260–0.825	0.009^*^
Skilled worker	0.549	0.320–0.939	0.029^*^
**Smoking by spouse/partner**			
No	1.000	Ref	Ref
Yes	2.457	0.946–6.382	0.065
**Use of illicit drugs by spouse/partner**			
No	1.000	Ref	Ref
Yes	6.358	1.452–27.836	0.014^*^
**History of psychiatric illness in spouse/partner**			
No	1.000	Ref	Ref
Yes	3.656	1.137–11.757	0.030^*^
**Spouse/partner suffered IPV in previous relationships**			
No	1.000	Ref	Ref
Yes	5.080	1.462–17.647	0.011^*^
**Spouse/partner witnessed parental IPV**			
No	1.000	Ref	Ref
Yes	9.342	4.448–19.619	< 0.001^*^

^*^Statistical significance

Women whose spouses/partners were professionals (OR = 0.463, 95% CI: 0.260–0.825, *p* = 0.009) or skilled workers (OR = 0.549, 95% CI: 0.320–0.939, *p* = 0.029) were less likely to experience IPV in pregnancy than those whose spouses/partners were unskilled/unemployed (Table 9). Women whose spouses/partners had history of psychiatric illness (OR = 3.656, 95% CI: 1.137–11.757, *p* = 0.030), whose spouses/partners had experienced IPV in previous relationship(s) (OR = 5.080, 95% CI: 1.462–17.647, *p* = 0.011), whose spouses/partners used illicit drugs (OR = 6.358, 95% CI: 1.452–27.836, *p* = 0.014), and those whose spouses/partners experienced parental IPV (OR = 9.342, 95% CI: 4.448–19.619, *p* < 0.001), were four, five, six, and nine times more likely to experience IPV in pregnancy compared to those whose spouses/partners had no history of psychiatric illness, had not experienced IPV in previous relationship(s), did not use illicit drugs and did not experience parental IPV, respectively (Table 9).

## Discussion

The prevalence of IPV in this study was 26.2%, with the most common type of IPV being psychological/emotional violence (24.6%), while sexual violence (8.1%) was the least reported type of IPV. Predictors of IPV included the women’s marital status (*p* = 0.001), educational status (*p* = 0.040), rural residence (*p* = 0.034), occupation (*p* = 0.040), spouse’s/partner’s occupation (p = 0.021), use of illicit drugs by spouse/partner (*p* = 0.014), history of psychiatric illness in spouse/partner (*p* = 0.030), experience of IPV in previous relationship(s) by spouse/partner (*p* = 0.011), and witness of parental IPV by spouse/partner (*p* < 0.001). The most common complication of IPV in pregnancy were the mental health complications of anxiety (54.7%) and depression (46.9%). Miscarriages (15.6%) and preterm birth (9.5%) were the most common pregnancy complications.

The overall IPV prevalence of 26.2% in this study approximates the World Health Organisation (WHO) reported global prevalence of IPV of 30%, but is lower than the reported IPV prevalence rate of 44% in SSA, with ranges from 13.9% in South Africa to as high as 97% in rural Nigeria. [4, 6, 17, 18]. We also found IPV prevalence rates of 27.7% and 24.4% in Nigeria and Ghana, respectively. The 2018 Nigeria Demographic and Health Survey (NDHS) revealed an IPV prevalence of 36% in Nigeria, while estimates from the 2016 national report on domestic violence in Ghana indicated that 27.7% of Ghanaian women have experienced IPV [19, 20].

The high rates of IPV reported in SSA is perpetuated by attitudes condoning domestic violence, as well as cultural norms that promote male dominance and control in the region. Evidence from the NDHS of 49 low- and middle-income countries shows that up to 79% of the population in SSA accept and justify domestic violence, with women even more likely than men to justify IPV [21]. In many African settings, wife beating is justified by both men and women as part of a normal intimate relationship and as punishment for women transgressing gender norms [22, 23]. In these settings, women are expected to be respectful and submissive to their husbands and revolting against or challenging domestic violence is frowned at, as the woman attempting to subvert the authority of the male partner [24]. Many women in SSA therefore do not report IPV, as seen in our study, where more than 50% of the women who experienced IPV never reported the violence to anyone, with two-third of them not reporting because they either did not want a third party to be aware or were afraid to report.

As similarly reported in previous studies in SSA [24, 25], sexual violence was the least type of IPV in our study. Given that most of the women in our study were married, this may not be unrelated to sociocultural norms in our setting that do not recognise the concept of marital rape/sexual violence in marriage [24]. It is generally believed culturally that once a man has married a woman, sex becomes the man’s right, without necessary need for consent from the woman [24].

Risk factors for IPV in pregnancy identified in our study, including low socioeconomic and educational status of the couple, rural residence, use of illicit drugs, history of psychiatric illness, experience of IPV and witness of parental IPV by spouse/partner, have been corroborated by other authors [6, 25–28]. Of every 10 pregnant women who had experienced IPV in this study, five each reported depression and anxiety, while almost 10% reported suicidal ideation/attempted suicide. Mental health disorders, including anxiety, depression, suicidal ideation and attempted suicide are significantly associated with IPV in pregnancy [12, 14, 29]. Mapayi et al. reported that women who had experienced IPV were 10 and 17 times, respectively, more likely to be depressed and anxious [30].

Miscarriages, reported by two of every 10 women who experienced IPV, and preterm birth, seen in 10% of the IPV victims, were the most common pregnancy complications of IPV in our study, while four and two women each reported IUFD/stillbirth and placental abruption, respectively. IPV has consistently been linked with adverse maternal and neonatal outcomes in pregnancy, including miscarriages, antepartum haemorrhage, preterm birth and perinatal deaths [12–14]. Berhanie et al. reported that pregnant women who experienced IPV were five times more likely to have adverse pregnancy outcomes and preterm delivery [11]. Preterm birth is a leading cause of neonatal mortality with significant long term neurological morbidity in survivors [31]. IPV is also negatively associated with initiation of antenatal care, number of antenatal care visits and utilisation of antenatal care services, and pregnant women who experience IPV are more likely to obtain antenatal care from unskilled providers, including traditional birth attendants [32]. All of these may have been contributory to the adverse pregnancy outcomes amongst women who experienced IPV in our study.

An adaptation of the HARK questionnaire was used for IPV screening in this study. Being a four-item screening tool requiring “YES” or “NO” answers, the HARK questionnaire is simple to score, easy to use and time-saving [16], especially in busy antenatal clinic settings like ours. The HARK questionnaire is easily self-administered by the many less literate subjects in our setting, hence its adoption in this study. This is in contrast to other screening tools like the revised conflict tactics scale (CTS2), which has 39 items with a total of 78 questions, which are scored on a Likert scale from 0 to 7, requiring 10–15 min to complete [33], which is not practicable in our busy antenatal clinics. Self-administration of the CTS2 by less literate subjects may be difficult; CTS was originally developed for interviewer-administration, requiring highly educated subjects for self-administration [34]. The HARK questions have been validated and extensively used to screen for IPV, with a sensitivity of 81% and specificity of 95% [15, 16].

The study findings have significant implications for healthcare workers and policy makers. Given that IPV is significantly associated with adverse pregnancy outcomes, we recommend that all pregnant women should be routinely screened for IPV during antenatal care in all clinical settings. This is currently not the case in many low-resource settings, including the two countries where our study was conducted. Routine screening for IPV in pregnancy will allow for early detection of IPV, intervention, and prompt referral to available support services. Such screening should identify screen-negative women who have the high-risk factors/predictors for IPV in pregnancy as identified in our study. These ones should be followed up closely for any signs of IPV. As part of antenatal care education, all pregnant women should be educated on IPV, its dangers, and the benefits of reporting early enough before complications set in. Government policies and interventions like free education, support for small and medium-sized enterprises, encouragement of female participation in politics and governance, and support of non-governmental organisations (NGOs) working on women’s rights and advocacy, will promote gender equality, women economic and educational empowerment. These, together with legislations that criminalise IPV and public enlightenment programmes on the dangers of IPV, smoking, alcohol consumption and use of illicit drugs, will reduce the prevalence of IPV as well as exposure of children to IPV at homes, a risk factor for IPV.

The strength of this study lies in its relatively large sample size compared to other studies, and its multicentre, binational study design. The use of a self-administered questionnaire may have allowed more women to report IPV, knowing that they are doing so confidentially. This is especially so in our setting, where many women do not report IPV, and are thus less likely to admit the experience of IPV if interviewed. However, an interviewer-administered questionnaire would have allowed us to identify and refer screen-positive women to support services. Most of our study respondents were urban dwellers, and it is well known (and as also corroborated by our study findings) that IPV occurs more frequently in rural settings [18, 24, 35]. The complications of anxiety and depression were based on self-reportage by the women and may be unreliable. Also, data on spousal experience of parental IPV and IPV in previous relationship were obtained by proxy from the women and may have been unreliable. Being a tertiary facility-based research, the findings of this study may not apply to the general population of pregnant women in both countries. These limitations notwithstanding, the study provides important data to guide policies and interventions to reduce the prevalence of IPV in pregnancy in both countries and the subregion. Future larger scale, highly powered, multi-country, population-based studies, with qualitative components, would provide more information on IPV in pregnancy that would be generalizable.

## Supplementary Information

Supplementary Material 1.

## Data Availability

The datasets used and analysed during the current study are available from the corresponding author on reasonable request.

## Abbreviations

CTS: Conflict tactics scale
CTS2: Revised conflict tactics scale
EGA: Estimated gestational age
HARK: Humiliation, Afraid, Rape, Kick
IPV: Intimate partner violence
IUFD: Intrauterine foetal death
SSA: Sub-Saharan Africa
