# Supplementary material for: Prevalence and determinants of intimate partner violence in pregnancy: a multicentre, binational study
Source: BMC Pregnancy Childbirth. 2025 Feb 5;25:124. doi: 10.1186/s12884-025-07177-z (PMC11800487; doi:10.1186/s12884-025-07177-z)
Supplement: Supplementary file 1 — Supplementary Material 1. [file 12884_2025_7177_MOESM1_ESM.docx]

**AN APPRAISAL OF INTIMATE PARTNER VIOLENCE IN PREGNANCY: A MULTICENTRE, BINATIONAL STUDY**

***QUESTIONNAIRE***

We are researchers from Nigeria and Ghana, currently investigating Intimate Partner Violence in Pregnancy. Intimate Partner Violence includes all forms of physical abuse, sexual abuse, controlling behaviours, and psychological aggression by a current or former partner or spouse.

Please kindly complete the following questionnaire; it will take less than 5 minutes. Participation in this study is voluntary, and you reserve the right to decline completing this questionnaire, or discontinue completing the questionnaire at any point. Your name, address, or any other identifying information are not required to fill this questionnaire; therefore, your responses are anonymous.

**CONSENT**:

Do you consent to participate in this study? Yes [ ] No [ ]

1. Age as at last birthday (in years)………………….
2. Marital status: Married [ ] Single [ ] Divorced [ ] Separated [ ] Co-habiting [ ]
3. Level of education: No formal education [ ] Primary [ ] Secondary [ ] Tertiary [ ]
4. Residence: Urban [ ] Rural [ ] Semi-urban [ ]
5. Geopolitical zone of residence: South-south [ ] South-west [ ] South-east [ ] North-west [ ] North-central [ ] North-west [ ]
6. Occupation: Professional, top civil servant, politician, businesswoman [ ] Middle-level bureaucrat, technician, skilled artisan, well to-do trader [ ] Unskilled worker and those in general whose income would be at or below the minimum wage [ ] Unemployed [ ]
7. Your spouse’s/partner’s/ex-spouse’s/ex-partner’s age as at last birthday (in years)……………………….
8. Your spouse’s/partner’s/ex-spouse’s/ex-partner’s level of education:

No formal education [ ] Primary [ ] Secondary [ ] Tertiary [ ]

1. Your spouse’s/partner’s/ex-spouse’s/ex-partner’s occupation:

Professional, top civil servant, politician, businesswoman [ ] Middle-level bureaucrat, technician, skilled artisan, well to-do trader [ ] Unskilled worker and those in general whose income would be at or below the minimum wage [ ] Unemployed [ ]

1. Religion: Christianity [ ] Islam [ ] African Traditional Religion [ ]

Others (please specifiy)…………………………………

1. How many times have you ever been pregnant? 0 [ ] 1 [ ] 2 [ ] 3 [ ] 4 [ ]

5 or more [ ]

1. How many spontaneous miscarriages have you had? 0 [ ] 1 [ ] 2 [ ] 3 [ ] 4 [ ]

5 or more [ ]

1. How many induced abortions/voluntary terminations of pregnancy have you had?

0 [ ] 1 [ ] 2 [ ] 3 [ ] 4 [ ] 5 or more [ ]

1. How many children do you have? 0 [ ] 1 [ ] 2 [ ] 3 [ ] 4 [ ] 5 or more [ ]
2. How many weeks pregnant are you?.......................................
3. Within the last 12 months (1 year), have you been humiliated or emotionally abused in other ways by your spouse/partner or ex-spouse/ex-partner?

Yes [ ] No [ ]

1. Within the last 12 months (1 year), have you been afraid of your spouse/partner or ex-spouse/ex-partner? Yes [ ] No [ ]
2. Within the last 12 months (1 year), have you been raped or forced to have any kind of sexual activity by your spouse/partner or ex-spouse/ex-partner?

Yes [ ] No [ ]

1. Within the last 12 months (1 year), have you been kicked, hit, slapped, or otherwise physically hurt by your spouse/partner/ex-spouse/ex-partner?

Yes [ ] No [ ]

1. During this pregnancy, have you been humiliated or emotionally abused in other ways by your spouse/partner or ex-spouse/ex-partner? Yes [ ] No [ ]
2. During this pregnancy, have you been afraid of your spouse/partner or ex-spouse/ex-partner? Yes [ ] No [ ]
3. During this pregnancy, have you been raped or forced to have any kind of sexual activity by your spouse/partner or ex-spouse/ex-partner? Yes [ ] No [ ]
4. During this pregnancy, have you been kicked, hit, slapped, or otherwise physically hurt by your spouse/partner or ex-spouse/ex-partner? Yes [ ] No [ ]
5. Does your spouse/partner/ex-spouse/ex-partner above, smoke cigarettes? Yes [ ] No [ ]

I don’t know [ ]

1. Does your spouse/partner/ex-spouse/ex-partner above, take illicit drugs (eg cocaine, marijuana, heroin, tramadol, codeine, etc)? Yes [ ] No [ ] I don’t know [ ]
2. Does your spouse/partner/ex-spouse/ex-partner above, have a family or personal history of psychiatric illness (eg depression, anxiety disorder, mania, bipolar disorder, psychotic disorder, schizophrenia etc)? Yes [ ] No [ ] I don’t know [ ]
3. a. Has your spouse/partner/ex-spouse/ex-partner above, been a victim of Intimate Partner Violence? Yes [ ] No [ ] I don’t know [ ]

b. Was your spouse/partner/ex-spouse/ex-partner brought up in a home/setting where he witnessed Intimate Partner Violence? Yes [ ] No [ ] I don’t know [ ]

1. Have you suffered any of the following complications/consequences of Intimate Partner Violence? (Please tick as many as applicable) Anxiety [ ] Depression [ ] Deliberate self-harm [ ] Suicidal ideation/attempt [ ] Panic disorder [ ] Divorce [ ]

Separation [ ] Marital disharmony [ ] Preterm (premature) labour [ ] Preterm (premature) delivery [ ] Prelabour (premature) rupture of foetal membranes (bag of water) [ ] Vaginal bleeding in pregnancy (threatened miscarriage) [ ] Premature separation of the placenta (abruptio placentae) [ ] Intrauterine foetal death/stillbirth [ ]

Low birthweight (less than 2.5kg) [ ] Spontaneous miscarriage [ ] Voluntary termination of pregnancy [ ] Others (please specify)……………………………………

1. If you have suffered any of the abuses above, did you report to anyone? Yes [ ] No [ ]
2. If Yes, to who did you report? The Police [ ] Social Welfare Department [ ]

Family/relatives [ ] Friends [ ] Others (please specify)……………………………..

1. If No, why did you not report? I was afraid [ ] I did not want a third party to be aware [ ] My spouse/partner/ex-spouse/ex-partner threatened me not to report [ ] No reason [ ] Others (please specify)……………………………………………………………….
